# In Vivo Cell Tracking Using PET: Opportunities and Challenges for Clinical Translation in Oncology

**DOI:** 10.3390/cancers13164042

**Published:** 2021-08-11

**Authors:** Laura M. Lechermann, Doreen Lau, Bala Attili, Luigi Aloj, Ferdia A. Gallagher

**Affiliations:** 1 Department of Radiology, University of Cambridge, Cambridge CB2 0QQ, UK; bala.attili@yahoo.com (B.A.); la398@cam.ac.uk (L.A.); fag1000@cam.ac.uk (F.A.G.); 2 Cancer Research UK Cambridge Centre, Cambridge CB2 0RE, UK; 3 Department of Nuclear Medicine, Addenbrooke’s Hospital, Cambridge CB2 0QQ, UK

**Keywords:** cell therapy, immunotherapy, cell tracking, PET/CT, PET/MRI, direct cell labelling, reporter genes, immuno-PET

## Abstract

**Simple Summary:**

Tracking therapeutic cells with non-invasive imaging methods has the potential to provide important information on the efficacy of cell therapies. In oncology, for example, monitoring the spatial distribution of chimeric antigen receptor (CAR) T-cells or tumour-infiltrating lymphocytes (TILs) could be used to monitor the efficiency of cellular trafficking to target sites within a patient. This review covers different cell labelling approaches for the non-invasive detection of therapeutic cells using positron emission tomography (PET). The potential for the clinical translation of these approaches and first-in-human studies is examined, as well as the translational challenges involved and how imaging can help overcome some of these challenges.

**Abstract:**

Cell therapy is a rapidly evolving field involving a wide spectrum of therapeutic cells for personalised medicine in cancer. In vivo imaging and tracking of cells can provide useful information for improving the accuracy, efficacy, and safety of cell therapies. This review focuses on radiopharmaceuticals for the non-invasive detection and tracking of therapeutic cells using positron emission tomography (PET). A range of approaches for imaging therapeutic cells is discussed: Direct ex vivo labelling of cells, in vivo indirect labelling of cells by utilising gene reporters, and detection of specific antigens expressed on the target cells using antibody-based radiopharmaceuticals (immuno-PET). This review examines the evaluation of PET imaging methods for therapeutic cell tracking in preclinical cancer models, their role in the translation into patients, first-in-human studies, as well as the translational challenges involved and how they can be overcome.

## 1. Introduction

Cell therapy is a rapidly evolving field and an important tool for personalised medicine in cancer. A wide spectrum of therapeutic cells coined as “living drugs” has been developed in recent years for the treatment of cancer, with many undergoing clinical trials, and some now licensed for clinical use. These include tumour-infiltrating lymphocytes (TILs), chimeric antigen receptor (CAR) T-cells, natural killer (NK) cells, dendritic cells, macrophage-based therapies, and drug-loaded neutrophils. CAR T-cells targeting CD19 (Kymriah^TM^ and Yescarta^TM^) are the first cell-based therapies to be approved by the United States Food and Drug Administration (FDA) for the treatment of acute lymphoblastic leukemia and diffuse large B-cell lymphoma, respectively. More recently, Tecartus^TM^, a CD20-directed CAR T-cell therapy was granted FDA approval for the treatment of adult patients with relapsed or refractory mantle cell lymphoma [1]. Novel technologies such as CRISPR-Cas9 gene-editing and bispecific CAR T-cell constructs have also been introduced in recent years to improve T-cell targeting and function within the tumour microenvironment [2,3].

As only a few are approved and clinically available, cell therapies largely remain at the research and translational phases, with safety and cost-benefit considerations representing some of the major challenges [4]. To successfully translate and clinically implement cell therapies, a better understanding of in vivo cellular behaviour is required, including biodistribution, tumour trafficking, tissue retention, and clearance. New tools for the optimisation of cell therapies are required to answer key questions, such as cellular localisation and accumulation at the target site, dynamic biodistribution, function, and viability of these cells over time in vivo, as well as the precise dosing, timing, and delivery of the administered cells to desired sites within the body.

In vivo imaging and tracking of cells can provide useful information for improving the accuracy and efficacy of cell therapies. Non-invasive imaging is ideal for the whole-body quantification and longitudinal monitoring of cellular and molecular processes. Cells can be labelled and tracked using a number of imaging modalities such as single-photon emission computed tomography (SPECT), positron emission tomography (PET), magnetic resonance imaging (MRI), and optical imaging [5]. White blood cell scintigraphy has been used for the imaging of infection and inflammation since the 1980s [6,7]. Autologous leukocytes are routinely labelled with lipophilic agents such as Technetium-99 m hexamethyl propylene amine oxime ([^99m^Tc]Tc-HMPAO), [^111^In]In-oxine, and [^111^In]In-tropolone [8,9]. Magnetic nanoparticles and fluorine-19 perfluorocarbon labelling of cells offers the opportunity to track cells without the use of ionising radiation but is limited by the low sensitivity of MRI and MR spectroscopy (MRS), as well as significant concentration of contrast agents that are required for detection [10,11]. Although cell tracking using optical imaging can provide valuable insights on single cell behaviour and cell-cell interactions at a microscopic level [12], the poor tissue penetrance of light and the limited spatial resolution of these techniques at a whole-body level, has limited the clinical application of optical imaging [13].

Positron emission tomography (PET), as a non-invasive imaging tool, has been successfully applied to tracking the spatio-temporal dynamics of administered therapeutic cells. PET is usually combined with computed tomography (PET/CT) to allow anatomical co-registration and attenuation correction of the detected photons for improved detection. PET/CT is widely available in most large hospitals and by far the most frequently used tracer is the glucose analogue [^18^F]-2-fluoro-2-deoxy-D-glucose ([^18^F]FDG), used to probe increased tumour metabolism for image-based treatment response assessment [14,15]. PET offers a very high sensitivity for cell tracking: Only a trace amount of the radiopharmaceutical in the order of picomolar concentrations is needed for detection, pharmacokinetic modelling, and determining the biodistribution of the administered activity [16,17]. Importantly, the measured PET signal on imaging is highly quantitative as individual counts can be directly related to the actual quantity of label, and simple reproducible metrics such as the standard uptake value (SUV), as well as tracer kinetics, can be used to provide quantitative measures of tracer uptake [18]. Recent development in total-body PET scanner technology to image the entire body has the potential to improve the sensitivity of detection by up to 40-fold for the whole body, and up to 5-fold for a single organ, and therefore offers a promising tool to quantifiably track a very small number of labelled therapeutic cells in vivo [19]. Furthermore, the use of PET to detect and track therapeutic cells has been facilitated by the development of new radiopharmaceuticals which offer a wide range of potential labels for cell labelling.

A number of different approaches can be used to label and image the spatial distribution of therapeutic cells in tissue: Direct labelling of cells ex vivo, indirect labelling of cells in vivo using gene reporters, as well as the detection of specific antigens expressed on the target cells using antibody-based radiopharmaceuticals (immuno-PET). This review focuses on PET radiopharmaceuticals for cell labelling strategies in preclinical models of cancer and the translation of these approaches into the clinic for therapeutic monitoring in patients. The merits and limitations of each strategy are discussed and examples of cell tracking approaches are demonstrated, highlighting the opportunities and challenges of clinical translation. The selection of the references was chosen to reflect a spectrum of applications and approaches, and was not based on defined search criteria.

## 2. In Vivo Imaging of Directly (Ex Vivo) Labelled Cells

### 2.1. Non-Metal Based PET Radioisotopes with a Short Half-Life

**Figure 1:**
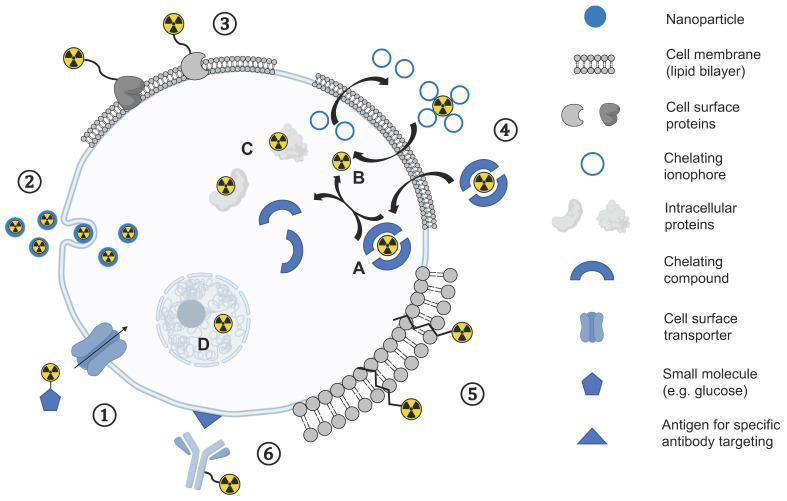
Overview of different direct cell labelling methods: **①** Uptake mediated by cellular transporters of small molecules (e.g., small molecules). **②** Endocytosis mediated uptake of nanoparticles. **③** Cell surface protein labelling. **④** Passive diffusion through the lipophilic cell membrane either as an intact chelate inside the cell (**A**) or dissociating in the cytosol (**B**) and association with intracellular proteins (**C**) or cell organelles (**D**). **⑤** Insertion of ligands into the lipophilic membrane. **⑥** Specific antigen targeting and subsequent internalisation of labelled antibody.

In a direct cell labelling approach, a tracer is added to cells ex vivo followed by incubation for cellular uptake of the label before labelled cells are injected into recipients. Different strategies for cell labelling using this approach have been explored using PET (Figure 1). Direct labelling of cells has been used for tracking the fate, biodistribution, and migration behaviour of autologous patient-derived cells following administration into patients using [^18^F]FDG ([^18^F], *t*_1/2_  =  109.7 min), the most frequently used and widely available clinical PET tracer in oncology, which is transported via glucose transporters (GLUT) and trapped intracellularly following phosphorylation by the enzyme hexokinase. [^18^F]FDG has been used to track the short-term delivery of cells in patients with cancer, inflammation, and infarction [20,21], and is more sensitive than the systemic administration of [^18^F]FDG given the high background levels of [^18^F]FDG uptake in many organs [22]. [^18^F]FDG has also been used for labelling genetically engineered NK-92-scFv(FRP5)-zeta natural killer cells in preclinical models: Higher uptake of NK-92-scFv(FRP5)-zeta cells were seen in HER2/neu-positive tumours within a 2-h time frame compared to the parental NK-92 cells and confirmed on autoradiography and histopathology [23]. For example, one study injected [^18^F]FDG-labelled macrophage-activated killer (MAK) cells into 10 patients with peritoneal relapse of epithelial ovarian carcinoma and showed a reproducible biodistribution pattern with trafficking to tumour sites up to 4 h following injection [24].

The use of shorter lived radiopharmaceuticals for cell tracking is more challenging, given the usual time taken for cells to migrate to the organ of interest. However, [^11^C]-methyl iodide ([^11^C], t_1/2_  =  20.3 min) has been used for the labelling of murine natural killer (NK) cells to measure the systemic distribution and short-term kinetics of tumour delivery in a fibrosarcoma model in mice [25]. Differences in the tumour accumulation between activated and non-activated cells were observed, as well as the cellular retention in the tumour up to 1 h post-injection. Despite promising initial imaging results, the relatively short half-lives of carbon-11 and fluorine-18, do not meet the requirements for longitudinal tracking of directly labelled cells over several days, which is the usual timescale for cellular migration. A general disadvantage of tracers targeting metabolic pathways is that they are taken up and trapped within cells by active transport or enzymatic activity. Therefore, labelling efficiency is heavily dependent on these functional processes which may vary between cells or within cells over time.

### 2.2. Metal-Based and Long-Lived PET Radioisotopes

Longer-lived PET isotopes offer the potential for tracking cells over several days: Copper-64 ([^64^Cu], *t*_1/2_  =  12.7 h) conjugated to nanoparticles has been investigated for the labelling of CD19-specific CAR T-cells using both superparamagnetic iron oxide nanoparticles (SPION) [26] and gold nanoparticles [27]. In a first-in-human study, CAR T-cells specific for the carbohydrate Lewis Y antigen were labelled ex vivo with ^64^Cu-labelled SPIONs, facilitated by a transfecting agent, and reinfused into patients with solid tumours to investigate the distribution of labelled cells to body organs and tumour sites within 3–5 days, using a hybrid PET/MRI approach [28]. [^64^Cu]Cu-pyruvaldehyde-bis(N4-methylthiosemicarbazone) ([^64^Cu]Cu-PTSM), a commonly used PET perfusion tracer, has also been used to study the biodistribution of labelled cells in healthy mice [29,30,31]. The lipophilic [^64^Cu]Cu-PTSM passively diffuses through the cell membrane and is trapped upon reduction inside the cell. However, the rapid cellular efflux of copper-64 has been shown to be a problem for long term cellular retention [31,32,33] and imaging is only possible for a few days [29,30,31]. Copper and also other metals such as manganese are essential metabolic elements with several key cellular roles, and therefore the maintenance of homeostasis can lead to low intracellular retention of these radiometals, which renders the PET isotopes of copper-64 and manganese-52 ([^52^Mn], *t*_1/2_  =  5.6 days) unsuitable for cellular labelling at later time points [34].

Successful cell tracking methods for routine clinical applications require long half-life tracers with more reliable labelling and cellular retention. Zirconium-89 ([^89^Zr], *t*_1/2_  =  78.4 h) has emerged as a promising PET radioisotope for direct cell labelling and has been widely used for antibody-based immuno-PET imaging over the last decade [35]. Analogous to [^111^In]In-oxine used in SPECT imaging, zirconium-89 can be used for the synthesis of the neutral and lipophilic complex [^89^Zr]Zr-oxine with four oxine (8-hydroxychinolin) ligands bound to zirconium-89 that enters cells through the same passive mechanisms [36]. Oxine acts as an ionophore that can transport radiometals across the cell membrane and as a meta-stable complex dissociates to deposit the radiometal inside the cell [34]. Zirconium-89 can bind to nuclear and cytoplasmic proteins (Figure 1), is biological inert, and is ideally suited for longitudinal cell imaging due to its residualizing properties within cells [35]. In recent years, [^89^Zr]Zr-oxine has been evaluated in several in vitro and preclinical in vivo studies for the labelling and tracking of therapeutic cells in preclinical oncology settings which are summarised in Table 1. This approach allows the imaging of labelled cells for at least 7 days post-injection due to the longer radioactive decay. The in vitro detection limit of [^89^Zr]Zr-oxine labelled T-cells has been found to be on the order of 10^4^ cells, with cellular activities as low as 15 kBq/10^6^ cells, on both clinical PET/CT and PET/MRI [37]. The clinical application of this approach is planned.

In contrast to the intracellular trapping of zirconium-89 using [^89^Zr]Zr-oxine, cell surface labelling with zirconium-89 can also be achieved via chelation with the use of desferrioxamine (DFO) based bifunctional chelators, such as the p-isothiocyanatobenzyl-linker (NCS), which forms amide bonds with primary amine groups on cell surface proteins. The stability of the [^89^Zr]Zr-DFO complex is of key importance since the release of free zirconium-89 results in non-specific uptake of zirconium-89 in vivo, such as in the bones. [^89^Zr]Zr-DFO-Bz-NCS has been used to label and image both CD19 targeting CAR expressing Jurkat cells and human T-cells in mouse xenograft CD19-positive models: Cells migrated from the lung to both the liver and spleen by day 1 and were detectable until day 7. However, no radioactive accumulation in CD19-positive tumours was observed [43].

Iodine-124 ([^124^I], *t*_1/2_  =  4.2 days) an organic PET isotope with one of the longest half -lives, has been used to label CD8^+^ T-cells specific for the ovalbumin peptide SIINFEKL [44]: 5-^124^I-iodo-2′-deoxyuridine ([^124^I]IUdR) is an analogue of thymidine, which labels proliferating cells by incorporation into DNA. Adoptively transferred ^124^I-labelled T-cells into mice with bilaterally implanted melanoma tumours showed higher signal accumulation in B16-OVA tumours expressing ovalbumin than the parental tumours (B16) on PET/MRI. This demonstrated the specificity of T-cell tumour targeting using this approach.

There are several important considerations for direct cell labelling approaches that need to be taken into account: The radiolabelling of cells can directly affect cellular function secondary to chemical alterations or radiation-induced changes, and it is important that labelled cells maintain viability and functionality post-labelling, as well as retain the radiolabel throughout the imaging time frame. Cellular efflux of the radiotracer or radioisotope in vivo can result in uptake and retention of free tracer in other tissues and organs resulting in non-specific background signal and therefore decreasing detectability of the injected cells of interest. Sensitivity for cell detection can be further compromised by the dilution of the signal from labelled cells due to cell proliferation over time, and therefore the detected radioactivity is not necessarily proportional to the number of labelled and injected cells.

## 3. In Vivo Imaging of Indirectly Labelled Cells Using Reporter Genes

**Figure 2:**
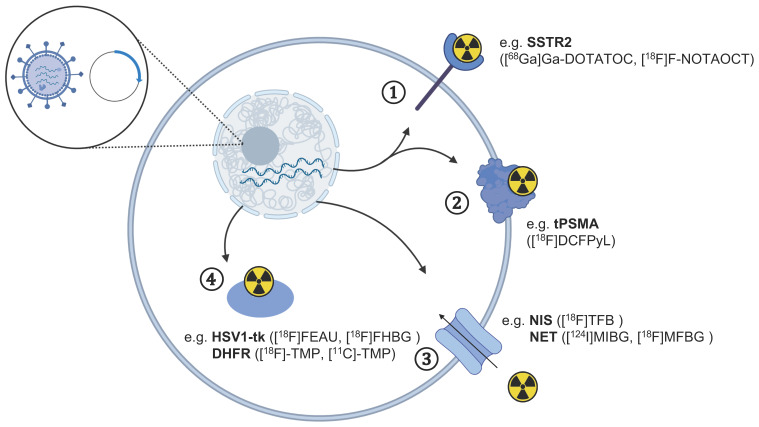
Overview of gene reporters for the tracking of therapeutic cells using different PET tracers. The transfection of cells with a vector plasmid leading to the expression of reporter genes in cells can be detected using PET imaging with radiotracers targeting specific **①** receptors, **②** cell surface enzymes, **③** protein transporters, and **④** intracellular enzymes. The respective PET tracer for each reporter gene discussed in the text is indicated in brackets. The figure was partly created with BioRender.com (accessed on 7 June 2021).

An additional strategy to track cells in vivo with radionuclide methods makes use of reporter genes. These are introduced in the desired cells to express targets that can be imaged with radiolabelled tracers after the injection of cells in vivo. This approach allows monitoring of therapeutic cells for an extended time with repeated tracer injection if adequate expression of the transduced gene is retained, addressing the limitations of radionuclide half-life, activity levels to inject, and the lower long-term stability of directly labelled cells. Figure 2 summarises the common reporter gene approaches for tracking CAR T-cells using different PET tracers with specific examples presented in Table 2.

### 3.1. Herpes Simplex Virus Thymidine Kinase (HSV1-tk)

**Figure 3:**
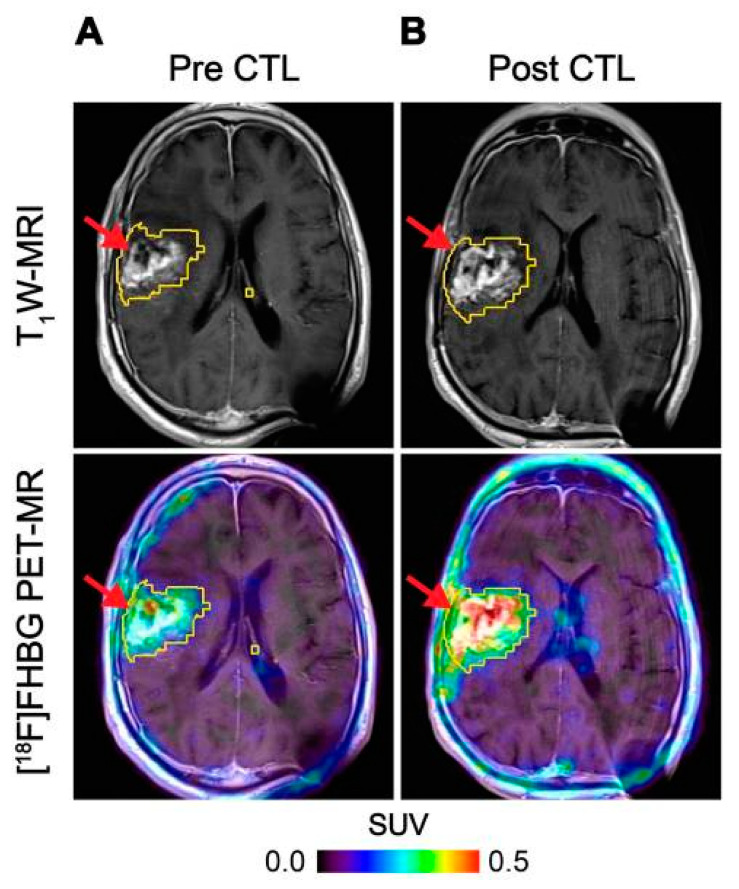
Imaging of HSV1-tk gene reporter expression in genetically modified CTLs with [^18^F]FHBG. [^18^F]FHBG-PET imaging was performed in a patient with recurrent glioblastoma multiforme tumour in the right frontoparietal lobe before (**A**) and 1 week after (**B**) CTL infusions. Allogeneic CTLs and IL-2 were injected intratumorally (red arrows). Tumour recurrence was monitored by T_1_-weighted (T_1_W) MRI (top panels) and [^18^F]FHBG-PET images were fused with the MR images (bottom panels). A significant increase in [^18^F]FHBG uptake and PET signal was detected following CTL infusion, which was likely to be due to CTL cell trafficking. Figure reproduced from [51].

HSV1-tk is an intracellular enzyme with high selectivity for the thymidine analogue ganciclovir, an inhibitor of viral DNA replication commonly prescribed to treat an HSV infection [56]. Radiolabelled ganciclovir analogues in combination with HSV1-tk expression have been utilised extensively as reporter systems for imaging [57]. The first human application of this approach used PET imaging with 9-[4-[^18^F]fluoro-3-(hydroxymethyl)butyl]guanine ([^18^F]FHBG) to track HSV1-tk transduced cytotoxic T-cells (CTLs) administered for treatment of recurrent high grade glioma [50]. The concept was expanded to monitor CTL trafficking, survival, and proliferation in seven patients with recurrent high-grade glioma that were resistant to conventional therapies by performing PET imaging with FHBG before and after intracerebral CTL infusion, where a significant increase in [^18^F]FHBG total activity was observed representing the CTL trafficking to tumours [51] (Figure 3). Therapeutic concentrations of ganciclovir analogues can be used to selectively kill HSV1-tk transduced cells. This additional feature provides a safety switch to stop the activity of the transferred cells if desired as shown in preclinical models [58,59], and may be particularly relevant in the regulation and control of CAR T-cells [60]. However, since HSV1-tk is a foreign protein and potentially immunogenic, there is concern that expression of this reporter gene may affect survival of the transduced cells once injected into patients as they may be vulnerable to T-cell mediated killing [61].

### 3.2. Somatostatin Receptor 2 (SSTR2)

Somatostatin receptor (SSTR) 2 is a G-protein-coupled cell surface receptor overexpressed in neuroendocrine tumours [62]. Several high affinity analogues of somatostatin have been developed as tracers and are routinely used for imaging SSTR2 overexpressing cancers in the clinical setting [63]. The most commonly utilised radiopharmaceutical for this purpose is the high affinity ligand [^68^Ga]Ga-DOTATOC [64] that has been explored in combination with SSTR2 for reporter gene imaging [65]. [^68^Ga]Ga-DOTATOC has been used to visualise the biodistribution and tumour infiltration of SSTR2-expressing human T-cells in a Jurkat cell murine tumour model [45]. The uptake of [^68^Ga]Ga-DOTATOC was shown to correlate with the tumour size and percentage of SSTR2-expressing T-cells. The very high affinity, rapid diffusion, and clearance of SSTR2 targeting PET tracers following injection, in addition to the location of the target on the cell surface, produces high target-to-background binding in a rapid timescale. Clinical imaging is usually performed 1 h after injection which provides optimal targeting [66], as opposed to the 2–3 h thus far used for HSV1-tk-based systems [50,67]. The ^18^F-labelled somatostatin analogue ^18^F-NOTA-octreotide (NOTAOCT) has been used to image SSTR-expressing neuroendocrine tumours in a clinical research study [68]. It has been successfully applied to track Intracellular Adhesion Molecule 1 (ICAM-1) targeting CAR T-cells transduced with SSTR2 with PET/CT in mice [46]. ^18^F-NOTA-octreotide-PET showed specific uptake by SSTR2-transduced CAR T-cells. The peak CAR-T cell signal was seen approximately 4 days following the peak tumour burden and gradually decreased to background levels thereafter.

SSTR2 is a human protein and thus poses no concern for immunogenicity of transduced cells. The use of this reporter system may be limited in the spleen as there is physiological expression of SSTR2 in haematopoietic cells causing very high specific binding in this organ. The very high level of SSTR2 expression in neuroendocrine tumours would not prevent this approach of being in these rare cancers. Since the physiological role of SSTR2 receptors is to exert inhibition on cell proliferation when activated, their expression in transduced cells does not pose concern for altering cell function. The activity of transduced cells may be controlled by the use of clinically approved drugs such as receptor agonists [69] or even with clinically approved radiolabelled drugs commonly utilised in the treatment of advanced neuroendocrine tumours [70].

### 3.3. The Sodium Iodide Symporter (NIS)

The human sodium iodine symporter (hNIS) is an additional reporter gene system that has been investigated for longitudinal cell tracking. The hNIS is physiologically expressed in thyroid, stomach, and salivary glands and drives uptake of the diagnostic and therapeutic radioactive isotopes of iodine and ^99m^Tc-pertechnetate ([^99m^Tc]TcO^4−^) into these cells. These radionuclides are clinically approved and commonly used for diagnostic studies and therapy of thyroid and salivary gland disorders. An experimental hNIS substrate, [^18^F]tetrafluorborate ([^18^F]TFB) [71,72], has been utilised in humans to image patients with thyroid cancer [73], as well as to image tumours and metastases in preclinical models [74,75]. The hNIS is an excellent candidate reporter gene for human application as it is non-immunogenic, is not internalized, and is only functional in viable cells [71]. [^18^F]TFB-PET has been used for quantifying differences in NIS-transduced CAR T-cell tumour retention in different triple-negative breast cancer models in mice with an impressive detection limit of ~3000 hNIS expressing cells [48]. Although the physiological uptake in normal hNIS expressing tissues is of concern, most of the background uptake with these tracers is in the head and neck region, allowing its use in other areas of the body. Several groups are validating this concept and clinical trials are in preparation.

### 3.4. Prostate Specific Membrane Antigen (PSMA)

PSMA is a cell-surface membrane glycoprotein that has been widely exploited for prostate cancer imaging. Novel ^68^Ga- and ^18^F-labelled small molecule PET ligands have been developed that can bind to PSMA with high selectivity and many of these are now in routine clinical application including the very recently FDA approved, radiofluorinated inhibitor of PSMA, 2-(3-{1-carboxy-5-[(6-[^18^F]fluoro-pyridine-3-carbonyl)-amino]-pentyl}-ureido)-pentanedioic acid ([^18^F]DCFPyL) [76]. Given the favourable pharmacokinetics and the very low expression in non-target organs, this system lends itself to applications for cell tracking. Anti-CD19 CAR T-cells expressing a N-terminal modified (N9del) PSMA, engineered to prevent internalisation and intracellular signalling which may affect cell physiology, have been tracked in a model of acute lymphoblastic leukaemia using [^18^F]DCFPyL [47]. PET imaging of CD19-tPSMA^(N9del)^ CAR T-cells showed very high sensitivity with a detection limit of ~2000 injected cells. Given the high sensitivity of this approach and the availability of therapeutic ligands of PSMA as safety switches, further development of this approach is appealing.

### 3.5. Norepinephrine Transporter (NET)

NET is a transmembrane protein expressed in the central nervous system, as well as in tumours of neural crest origin. It mediates the transport of norepinephrine, dopamine, and epinephrine across the cell membrane [77]. Radiolabelled derivatives of norepinephrine are clinically available for imaging phaeochromocytoma, paraganglioma, and neuroblastoma. Metaiodobenzyl-guanidine (MIBG) labelled with iodine-131 or iodine-123 are routinely used for imaging and therapy of advanced cancers. The development of ^18^F-labelled metafluorobenzyl-guanidine ([^18^F]MFBG), as well as the use of ^124^I-metaiodobenzylguanidine ([^124^I]MIBG), have opened up the use of PET for this approach. The hNET has also been used as a reporter gene to image transduced T-cells in mice with [^124^I]MIBG and [^18^F]MFBG with the latter offering the higher sensitivity in the range of 3–4 × 10^4^ T-cells at the site of injection [49]. However, clinical translation of this approach would require a significantly higher tumour uptake compared to the endogenous NET expression in organs with sympathetic innervation, which demonstrates a significant background signal [78].

### 3.6. E. coli Dihydrofolate Reductase (eDHFR)

More recently, an approach based on the bacterial enzyme dihydrofolate reductase derived from *E. coli* (eDHFR) has been used as a reporter gene for cellular tracking. The eDHFR can be imaged using radiolabelled versions of its ligand, i.e., a derivative of the antibiotic trimethoprim (TMP). TMP has been labelled with carbon-11 ([^11^C]-TMP) [54] and more recently with fluorine-18 ([^18^F]-TMP) for studying the trafficking of anti-GD2 CAR T-cells in NOD scid gamma (NSG) mouse mice bearing GD2^+^ human osteosarcoma xenografts [55]. Radiolabelled TMP has the potential to achieve low background in unmodified mammalian tissues and high retention in eDHFR engineered cells. [^18^F]-TMP provided high contrast imaging with a detection sensitivity of ∼11,000 cells per mm^3^. The eDHFR system does not provide a readily available safety switch and requires stable expression of a bacterial protein, which is less desirable than the expression of a human protein.

## 4. In Vivo Imaging of Cells Using Antibodies (Immuno-PET)

Therapeutic cells can also be imaged in vivo using radiolabelled antibodies and antibody fragments. Indirect labelling of cells with antibodies provides high affinity and specificity for the target. A number of established conjugation strategies and radiolabelling approaches can be used for synthesising antibody-based imaging probes [79]. Cell surface markers (such as CD3, CD4, and CD8), as well as activation or exhaustion markers (such as OX40 and PD-1), can be detected non-invasively using radiolabelled antibodies to determine the functional status of the administered cells (Table 3). The density or number of labelled cells at the tumour sites can be longitudinally tracked using repeated injections overcoming the problems with signal dilution following a single injection [80].

Antibody labelling may produce functional effects on the therapeutic cells, which require evaluation both in vitro and in vivo [79]. For example, the presence of an intact Fc region in certain clones of antibodies has been shown to deplete target cells and this can be overcome by engineering antibodies with cleaved Fc region or antibody fragments without the Fc receptor [79,81]. Unmodified antibodies (~150 kDa) can exhibit slow clearance due to the interaction with the neonatal Fc receptor and a prolonged serum half-life: Imaging at earlier time points can be problematic due to the high background signal and non-specific tissue accumulation. To circumvent this, enzymatic or genetic modifications of antibodies have been used to engineer smaller antibody derivatives or fragments (~25–100 kDa) such as: Monovalent F(ab’) and divalent F(ab’)^2^ with the antigen-binding regions linked by disulfide bonds and no Fc portion; single-chain variable fragments (scFv); minibodies; diabodies; and even smaller therapeutic proteins (<6 KDa), such as affibodies and nanobodies [79]. Each of these antibody derivatives exhibit different pharmacokinetics and clearance properties. Smaller fragments with sizes below the renal filtration threshold (<60 kDa) are generally excreted through the kidneys and have a rapid blood clearance with minimal background signal, while full-length and larger antibody derivatives are cleared through the hepatobiliary route. However, there is often a trade-off between maximum target accumulation and minimal background signal [81]. Larger molecules tend to accumulate non-specifically in solid tumours due to the enhanced permeability and retention (EPR) effects, as tumours tend to have irregular blood vasculature and inefficient lymphatic drainage [82]. Therefore, when designing antibody-based radiopharmaceuticals, it is very important to distinguish non-specific tumour uptake from true target engagement.

The choice of antibody conjugation strategy is also important when designing radiopharmaceuticals to detect therapeutic cells [83]. Ideally, chemical modifications should not compromise the functionality and binding affinity of an antibody to its target, and the reactions should occur under mild conditions to minimize protein denaturation. Bifunctional chelators are often employed as a link between the antibody and a metal-based radionuclide [84]. Examples include diethylenetriamine pentaacetate (DTPA), tris(hydroxypyridinone) (THP), and desferrioxamine (DFO) that can be radiolabelled under ambient temperature and mild acidic to neutral pH [84]. The choice of radionuclide should ideally match the biological half-life (t_1/2_) of the molecules. Short-lived radionuclides such as Gallium-68 are suitable for scFv and diabodies with rapid blood clearance, whilst a long-lived positron emitter such as Zirconium-89 is more suitable for detecting therapeutic cells with full-length antibodies [85].

**Figure 4:**
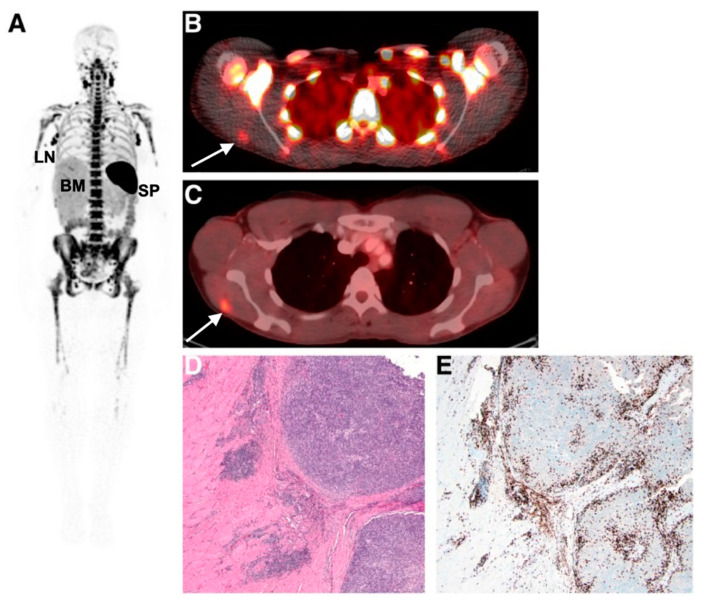
Imaging human T-cell tumour infiltration using [^89^Zr]Zr-IAB22M2C, a radiolabelled minibody targeting CD8. (**A**) Normal splenic, bone marrow, and lymph node uptake of the tracer were observed. Uptake of (**B**) [^89^Zr]Zr-IAB22M2C and (**C**) [^18^F]FDG in a melanoma metastasis in the deltoid muscle of a patient was histologically confirmed as high CD8^+^ T-cell infiltration (**D**,**E**). Figure reproduced from [87].

Imaging and detecting the presence of therapeutic T-cells in tumours has been demonstrated using CD8 antibody fragments (Figure 4) [86,87,88]. For example, CD8 cys-diabodies ([^89^Zr]Zr-malDFO-169 cDb) engineered from rat hybridoma cell lines have been used for the non-invasive tracking of cytotoxic T-cells in murine models of cancer immunotherapy [86]. A higher uptake of the tracer was detected in ovalbumin-expressing tumours following adoptive transfer of CD8^+^ T-cells expressing the MHC Class I-restricted TCR specific for ovalbumin (Ova) [86]. Preliminary data from a first-in-human study on six patients using [^89^Zr]Zr-IAB22M2C, a radiolabelled minibody targeting CD8, demonstrated favourable pharmacokinetics and a good safety profile [87]. Tracer uptake in CD8^+^ T-cell-rich tissues such as the lymph nodes was seen as early as 2 h post-injection. Tumour uptake was noted in two patients receiving immunotherapy (metastatic melanoma and hepatocellular carcinoma), but not in the remaining four patients with lung metastases. The tumour uptake of [^89^Zr]Zr-IAB22M2C was also histologically confirmed as CD8^+^ T-cell infiltration at the periphery of a metastatic lesion in the deltoid muscle of a patient with melanoma. The uptake of [^89^Zr]Zr-IAB22M2C in tumours showed areas of both concordance and discordance with [^18^F]FDG-PET uptake, in addition to non-specific uptake in bone marrow and lymph nodes. Therefore, [^89^Zr]Zr-IAB22M2C can be used as a complementary tracer to [^18^F]FDG for the direct imaging of CD8^+^ T-cells.

Mall et al. developed [^89^Zr]Zr-Df-aTCRmu-F(ab’)_2_ specific for the murine T-cell receptor (TCR) beta domain to track transgenic human T-cells engineered with murine sequences in the TCR [89]. Differential patterns of distribution of [^89^Zr]Zr-aTCRmu-F(ab’)_2_ signals were detected in the tumours, i.e., larger tumours exhibited intense signals at the tumour border, whilst smaller tumours demonstrated uniform distribution of signals. The sensitivity and clinical applicability of the method was further evaluated in a separate study [90], whereby a detection limit of 1.0 × 10^4^ T-cells was observed when imaging was performed on tumour-bearing NSG mice injected with different numbers of transgenic T-cells. This observation at the preclinical level is promising and compatible with the number of human CAR T-cells usually administered into patients for treatment in clinical trials, which is of the order of 10^4^ to 10^8^ per kilogram body weight [91].

Radiolabelled antibodies can also be used for monitoring the efficacy of NK cell therapies. A recent example is the use of radiolabelled IgG1 antibodies specific for the human NK cell activation receptor NKp30, i.e., [^64^Cu]Cu-NKp30Ab to detect NK cell trafficking in an adoptive cell transfer model [92]. A high specific uptake of the tracer was demonstrated in vitro on the human NK cell line NK92MI and human NK cells isolated from buffy coats and in vivo on NKp30-expressing xenografts. The specific detection of human NK cells residing in the liver and spleen of NSG mice following adoptive cell transfer further demonstrated the clinical feasibility of this approach.

Determining the functional status of therapeutic cells over the course of treatment is important to ensure treatment efficacy. Radiolabelled antibodies targeting activation and exhaustion markers on therapeutic cells have been developed for oncological applications. Expression of the T-cell activation marker, inducible T-cell costimulatory receptor (ICOS) was evaluated using a full-length ICOS antibody, [^89^Zr]Zr-DFO-ICOS, 5 days after CD19-specific T-cell administration in a murine model of B-cell lymphoma [93]. Although specific uptake of the tracer seen in the bones correlated with the presence of CAR T-cells infiltrating B-cell lymphoma in the bone marrow, using a full-length antibody for imaging ICOS was limited as a substantial amount of non-specific uptake was observed in highly vascularized organs such as the heart, liver, and spleen. Thus, further modification of the ICOS antibody into more miniaturized forms may be needed to improve the pharmacokinetics of this tracer for clinical translation.

The expression of immune checkpoint proteins on tumour-infiltrating lymphocytes and CAR T-cells is a strong indication of immune tolerance and exhaustion, and a hallmark of treatment failure [94]. Monitoring the expression of immune checkpoint proteins on therapeutic T-cells and in the tumour microenvironment is important for determining long-term treatment efficacy. Notable examples of radiopharmaceuticals that are at the stage of preclinical and clinical testing include radiolabelled antibodies targeting the programmed cell death 1 receptor (PD-1) [^89^Zr]Zr-nivolumab and [^89^Zr]Zr-pembrolizumab, its ligand PD-L1 [^89^Zr]Zr-atezolizumab, and [^18^F]F-BMS-986192, as well as the lymphocyte-activation gene 3 (LAG-3) specific [^89^Zr]Zr-DFO-REGN3767 [95,96,97,98].

[^89^Zr]Zr-nivolumab has been investigated both in a humanised mouse model of lung cancer [99], as well as in patients with non-small cell lung carcinoma [96]. The tracer showed high specific binding to PD-1 expressing T-cells both in vitro and in vivo, and was associated with T-cell infiltration in the tumours, salivary, and lacrimal glands of NSG mice engrafted with human peripheral blood mononuclear cells [99]. In patient studies, a high tracer accumulation was observed in the spleen, which is likely to be due to interactions with PD-1 expressed on lymphocytes and dendritic cells [96]. [^89^Zr]Zr-nivolumab uptake in tumours was histologically confirmed as PD-1^+^ T-cells and was predictive of response to the nivolumab treatment. A competitive antagonist of PD-L1 has been radiolabelled with copper-64 for imaging PD-L1 expression in mice: [^64^Cu]Cu-DOTA-HAC [100]. This radiotracer was based on a soluble fragment of the PD-1 ectodomain: It exhibits a high affinity for PD-L1 (110 pM), is small in size (14 kDa), and does not contain an F_c_ region, thus avoiding the intrinsic limitation of antibodies as discussed above. A high tumour uptake and favourable tumour-to-background ratios were observed at 1 h post-injection of [^64^Cu]Cu-DOTA-HAC. The radiotracer was shown to be highly specific for human PD-L1 and persisted in PD-L1+ tumours for at least 24 h. The rapid and specific uptake of [^64^Cu]Cu-DOTA-HAC shows promise for further evaluation in clinical trials.

## 5. Conclusions and Perspectives

Imaging of cell-based cancer immunotherapies including genetically engineered cells has found an important role in basic cancer research and is becoming a valuable tool for the translation of new cell therapies into clinical settings. The ability to follow the biodistribution of these cells in vivo provides important information on whether target engagement has been successful, the intratumoural and intermetastatic heterogeneity of therapeutic cell delivery, and how the cell uptake changes longitudinally. These data could help predict and stratify which patients will respond to therapy as part of a personalised treatment and could also be used to detect early response to therapy before changes in tumour size are apparent. In this way, labelling a small percentage of the injected therapeutic cell population could act as a companion biomarker for the larger proportion of cells used for the treatment. Cell labelling methods have a wide range of applications in addition to their use in oncology, and these approaches could be of great value for labelling stem cells or other cell therapies in neurological and autoimmune diseases, as well as for studying infectious diseases.

Imaging allows longitudinal tracking of therapeutic cells within a patient to be undertaken non-invasively, as well as the detection of tumour heterogeneity, which is more difficult with competing approaches such as tissue biopsy or liquid biomarkers. PET affords a very high sensitivity for the detection of radiolabelled cells, and can report on cell tracking at high spatial and temporal resolution. The spatial resolution of the radiolabel within the tumour is limited by the fundamental PET resolution determined by the mean distance travelled by a positron before annihilation, which varies with positron energy and is isotope-specific, e.g., 0.6 mm for fluorine-18, 1.2 mm for zirconium-89, and 2.9 mm for gallium-68 [107]. In practice, the achievable spatial resolution is lower and usually of the order of several millimetres for most clinical PET applications. Temporal resolution is limited by the number of counts acquired within a given time window to ensure that the signal from the tumour or organ can be discriminated from background or noise. The required temporal resolution for monitoring cell influx and efflux is of the order of hours to days and is therefore not limited by the temporal resolution of the scanner, but rather by the loss of signal due to either the isotope half-life or from label dilution due to cellular proliferation for the direct cell labelling approaches. In practice, this is limited to 7–10 days for long half-live radionuclides but could be extended in the future with the increased sensitivity that will be afforded by total body PET systems [19].

The approaches to cell labelling described in this review provide complementary information: Some assess the resident tumour immune populations, while others report on the trafficking of cells in or out of the tumour. Direct ex vivo cell labelling specifically shows the distribution of the injected labelled population and how it is taken up into the tumour or organ of interest, with little or no background signal to complicate the analysis. A potential complication of all cell labelling approaches, including direct cell labelling, is that some of the labels could be released and may subsequently accumulate in adjacent cellular subpopulations. Antibody or antibody fragment labelling is also highly specific, albeit for a target rather than a cell population, so may label more than one resident cell population and will demonstrate some non-specific background accumulation which may reduce the sensitivity for detection. Antibody labelling also has the benefit of providing functional information in addition to spatial localization and can inform on cellular activation status and cell-cell interactions. Reporter genes are the most attractive approach given their potential for a very high level of cellular specificity and since the target is not diluted with cellular proliferation or tumour metastasis. The choice of reporter gene/target is based on multiple factors: (a) The availability of specific tracers which ideally are suitable for PET and clinically approved; (b) the background expression in tumours or normal organs, which ideally is as low as possible; (c) favourable dosimetry to minimise concerns over radiation exposure, and allow for measurement at multiple time points after administration of the tagged cells; (d) limited or no biological effect deriving from expression of the transgene; (e) alternatively, a transgene may be chosen to serve as a therapeutic effector or target for its application. The potential for non-human reporter genes to be immunogenic must also be taken into consideration when addressing ideal system design, since this may affect functionality and survival of transduced cells once injected into humans.

Two or more of these approaches could be combined using isotopes with different half-lives to provide a multiparametric readout of both the resident immune cells, as well as influx of cells from the circulation, i.e., a dual-isotope imaging approach. Alternatively, a PET label could be incorporated into an experimental bifunctional probe, using MRI-based approaches, for example, to probe more than one cellular population simultaneously or to provide complementary simultaneous readouts as part of hybrid imaging with PET/MRI.

For clinical translation of PET cell labelling to be more widely used, significant technical and regulatory hurdles need to be overcome. SPECT cell labelling is already part of clinical routine, and therefore good manufacturing practice (GMP) approaches and the required infrastructure required for radiolabelling of cells already exists in many larger institutions. The radiochemistry synthesis involved may have to be upscaled so that it can be stably reproduced for routine large scale clinical use. CAR T-cell manufacturing processes are well established and therefore the addition of PET labelling as a companion biomarker for these therapies which already have obtained regulatory approval, would require a change in practice and new approvals. As the PET label is found in trace quantities, most of the labelling approaches described here do not require additional toxicology assessment when an established PET label is conjugated to human cells. However, if there is a possibility of probe-target interaction resulting in deleterious effects on cell function and viability, specific toxicology studies may be required in some instances.

The introduction of a transgene into live cells for human administration can raise safety concerns and requires extensive regulatory scrutiny and validation before it can be considered for clinical use to ensure long term stability and safety. In addition, preparation of transduced cells for human injection is significantly more complicated and expensive than the requirements for direct cell labelling procedures. Although reporter genes pose significant challenges before being used routinely in a clinical setting, they present many benefits when this can be achieved.

A key element in the translation of these techniques is clinical acceptance and evidence of utility in a clinical setting. The imaging of cell therapies is a relatively new area and a regulatory framework for more routine imaging studies remains to be defined. Most studies to date have involved small numbers of patients from a single institution. Future larger multisite studies are required to provide the evidence for both regulators and clinicians, and it will be important to engage early with the pharmaceutical industry when designing these studies. Repeatability and reproducibility are also key steps in the technical validation stage of these studies. In the longer term, if cell labelling can be shown to better stratify expensive cell therapies, then the imaging costs can be defrayed by reducing the use of ineffective treatments or replacing them with more effective therapies at earlier timepoints. This will provide evidence to deliver a change in clinical practice, and education and training of both the imaging and oncological communities will facilitate this. In conclusion, in vivo cell labelling using PET is a promising area for improving the understanding of tumour biology, as well as addressing important clinical questions in the emerging field of cell therapies.

## Figures and Tables

**Table 1:** Preclinical cell labelling and imaging studies using 10^6^–10^7^ cells labelled with [^89^Zr]Zr-oxine.

Cell Type	Purpose	Cellular Activity [kBq/10^6^]	Imaging [Time]	Reference
Dendritic cells (DCs), activated cytotoxic T-cells (CTLs),	In vitro labelling study and in vivo tracking of CTLs to B16-OVA melanoma tumours	89–111 (DCs) 37 (CTLs)	7 days	[38]
Mesenchymal Stem cells (MSCs) transduced to express TNF-related apoptosis inducing ligand (TRAIL)	In vivo tracking and biodistribution of MSC-TRAILs to mesothelioma tumours	311	7 days	[39]
Natural killer cells	In vivo tracking and biodistribution in healthy rhesus macaques	13.7 ± 5.2	7 days	[40]
Vγ9Vδ2 subtype of human γδ T-cells	In vivo tracking of γδ T-cells in a xenograft model of breast cancer	30–300	7 days	[41]
Human CD19^+^ CAR T-cells	Trafficking of intraventricular injected IL13Rα2 CAR T-cells to tumour sites	70	7 days	[42]

**Table 2:** Summary of reporter genes used for the imaging of CAR T-cells in cancer.

Reporter Gene	Tracer	Cell Type/Model	Detection Limit	Reference
***hSSTR2***	[^68^Ga]Ga-DOTATOC, [^18^F]F-NOTAOCT	ICAM-1 specific CAR T-cells; Jurkat T-cells	4 × 10^6^ cells/cm^3^ in vivo	[45,46]
***tPSMA^N9de^***	[^18^F]DCFPyL	CD19-tPSMA^(N9del)^ CAR T-cells	2 × 10^3^ cells in vitro	[47]
***NIS***	[^18^F]TFB	T4NT CAR T-cells	3 × 10^3^ cells in vitro	[48]
***hNET***	[^124^I]MIBG, [^18^F]MFBG	Comparative study with transduced T-cells	3–4 × 10^4^ cells in vivo	[49]
***HSV1-tk***	[^18^F]FEAU, [^18^F]FHBG	IL-13 zetakine CAR T-cells, CD19 CAR T-cells, hPSMA specific CAR T-cells	~3 × 10^5^ cells in vivo	[50,51,52,53]
***eDHFR***	[^18^F]-TMP, [^11^C]-TMP	GD2^+^ CAR T-cells	4 × 10^6^ cells/1 cm^3^ in vitro	[54,55]

**Table 3:** Tracking therapeutic cell location and functional status using radiolabelled antibodies.

Target	Radiopharmaceutical	Imaging	Reference	Stage of Development
**Cell Surface Markers**				
CD3	[^89^Zr]Zr-DFO-CD3	CD3 cell infiltration (after anti-CTLA-4 treatment)	[101,102]	Preclinical
CD4	[^89^Zr]Zr-DFO-CD4	Whole body assessment of CD4 status to predict response to checkpoint inhibition	[103]	Preclinical
CD8	[^89^Zr]Zr-malDFO-169 cDb [^89^Zr]Zr-Df-IAB22M2C	CD8 T-cell distribution/infiltration and changes in response to immunotherapy	[86,87,104]	Preclinical Clinical *NCT03802123*
TCR	[^89^Zr]Zr-Df-aTCRmu-F(ab’)_2_	T-cell receptor	[90]	Preclinical
**Activation Markers**				
OX40	[^64^Cu]Cu-DOTA-AbOX40	Detection of T-cell activation to predict tumour response to vaccine	[105]	Preclinical
ICOS	[^89^Zr]Zr-DFO-ICOS	Noninvasive tracking of murine CD19–28z CAR-T cells in B-cell lymphoma	[93]	Preclinical
NKp30	[^64^Cu]Cu-NKp30Ab	Tumour-infiltrating NK cells and adoptive transfer of NK cells for therapy	[92]	Preclinical
**Exhaustion Markers**				
CTLA-4	[^64^Cu]Cu-NOTA-ipilimumab-F(ab’)_2_	Expression of CTLA-4 on tumour-infiltrating lymphocytes	[106]	Preclinical
PD-1	[^89^Zr]Zr-nivolumab	Expression of PD-1 on tumour-infiltrating lymphocytes	[96,98]	Clinical *NCT03843515*
	[^89^Zr]Zr-pembrolizumab			
PD-L1	[^89^Zr]Zr-atezolizumab	Expression of PD-L1 on tumour microenvironment	[95,96,100]	Clinical
	[^18^F]F-BMS-986192			*NCT04222426*
	[^64^Cu]Cu-DOTA-HAC			*NCT03843515*
LAG-3	[^89^Zr]Zr-DFO-REGN3767	Expression of LAG-3 on tumour-infiltrating lymphocytes	[97]	Clinical *NCT04706715*

## Data Availability

Not applicable.

## References

[1] Wang M., Munoz J., Goy A., Locke F.L., Jacobson C.A., Hill B.T., Timmerman J.M., Holmes H., Jaglowski S., Flinn I.W. (2020). KTE-X19 CAR T-Cell Therapy in Relapsed or Refractory Mantle-Cell Lymphoma. N. Engl. J. Med..

[2] Shah N.N., Johnson B.D., Schneider D., Zhu F., Szabo A., Keever-Taylor C.A., Krueger W., Worden A.A., Kadan M.J., Yim S. (2020). Bispecific anti-CD20, anti-CD19 CAR T cells for relapsed B cell malignancies: A phase 1 dose escalation and expansion trial. Nat. Med..

[3] Roth T.L., Puig-Saus C., Yu R., Shifrut E., Carnevale J., Li P.J., Hiatt J., Saco J., Krystofinski P., Li H. (2018). Reprogramming human T cell function and specificity with non-viral genome targeting. Nature.

[4] Lin J.K., Muffly L.S., Spinner M.A., Barnes J.I., Owens D.K., Goldhaber-Fiebert J.D. (2019). Cost Effectiveness of Chimeric Antigen Receptor T-Cell Therapy in Multiply Relapsed or Refractory Adult Large B-Cell Lymphoma. J. Clin. Oncol..

[5] Kircher M.F., Gambhir S.S., Grimm J. (2011). Noninvasive cell-tracking methods. Nat. Rev. Clin. Oncol..

[6] McAfee J.G., Samin A. (1985). In-111 labeled leukocytes: A review of problems in image interpretation. Radiology.

[7] Palestro C.J., Love C., Bhargava K.K. (2009). Labeled leukocyte imaging: Current status and future directions. Q. J. Nucl. Med. Mol. Imaging.

[8] De Vries E.F.J., Roca M., Jamar F., Israel O., Signore A. (2010). Guidelines for the labelling of leucocytes with 99mTc-HMPAO. Eur. J. Nucl. Med. Mol. Imaging.

[9] Roca M., de Vries E., Jamar F., Israel O., Signore A. (2010). Guidelines for the labelling of leucocytes with 111 In-oxine. Eur. J. Nucl. Med. Mol. Imaging.

[10] Fink C., Gaudet J.M., Fox M.S., Bhatt S., Viswanathan S., Smith M., Chin J., Foster P.J., Dekaban G.A. (2018). ^19^F-perfluorocarbon-labeled human peripheral blood mononuclear cells can be detected in vivo using clinical MRI parameters in a therapeutic cell setting. Sci. Rep..

[11] De Vries I.J.M., Lesterhuis W.J., Barentsz J.O., Verdijk P., Van Krieken J.H., Boerman O.C., Oyen W.J.G., Bonenkamp J.J., Boezeman J.B., Adema G.J. (2005). Magnetic resonance tracking of dendritic cells in melanoma patients for monitoring of cellular therapy. Nat. Biotechnol..

[12] Lau D., Garçon F., Chandra A., Lechermann L.M., Aloj L., Chilvers E.R., Corrie P.G., Okkenhaug K., Gallagher F.A. (2020). Intravital Imaging of Adoptive T-Cell Morphology, Mobility and Trafficking Following Immune Checkpoint Inhibition in a Mouse Melanoma Model. Front. Immunol..

[13] Hillman E.M.C., Amoozegar C.B., Wang T., McCaslin A.F.H., Bouchard M.B., Mansfield J., Levenson R.M. (2011). In vivo optical imaging and dynamic contrast methods for biomedical research. Philos. Trans. R. Soc. A Math. Phys. Eng. Sci..

[14] Mankoff D.A., Katz S. (2018). PET imaging for assessing tumor response to therapy. J. Surg. Oncol..

[15] Wahl R.L., Jacene H., Kasamon Y., Lodge M.A. (2009). From RECIST to PERCIST: Evolving Considerations for PET Response Criteria in Solid Tumors. J. Nucl. Med..

[16] Rahmim A., Zaidi H. (2008). PET versus SPECT: Strengths, limitations and challenges. Nucl. Med. Commun..

[17] Rodriguez-Porcel M. (2010). In Vivo Imaging and Monitoring of Transplanted Stem Cells: Clinical Applications. Curr. Cardiol. Rep..

[18] Zaidi H., Karakatsanis N. (2018). Nuclear medicine: Physics special feature review article: Towards enhanced pet quantification in clinical oncology. Br. J. Radiol..

[19] Cherry S.R., Jones T., Karp J.S., Qi J., Moses W.W., Badawi R.D. (2018). Total-body PET: Maximizing sensitivity to create new opportunities for clinical research and patient care. J. Nucl. Med..

[20] Bhattacharya A., Kochhar R., Sharma S., Ray P., Kalra N., Khandelwal N., Mittal B.R. (2014). PET/CT with ^18^F-FDG-Labeled Autologous Leukocytes for the Diagnosis of Infected Fluid Collections in Acute Pancreatitis. J. Nucl. Med..

[21] Hofmann M., Wollert K.C., Meyer G.P., Menke A., Arseniev L., Hertenstein B., Ganser A., Knapp W.H., Drexler H. (2005). Monitoring of Bone Marrow Cell Homing into the Infarcted Human Myocardium. Circulation.

[22] Wu C., Ma G., Li J., Zheng K., Dang Y., Shi X., Sun Y., Li F., Zhu Z. (2013). In vivo cell tracking via ^18^F-fluorodeoxyglucose labeling: A review of the preclinical and clinical applications in cell-based diagnosis and therapy. Clin. Imaging.

[23] Meier R., Piert M., Piontek G., Rudelius M., Oostendorp R.A., Senekowitsch-Schmidtke R., Henning T.D., Wels W.S., Uherek C., Rummeny E.J. (2008). Tracking of [^18^F]FDG-labeled natural killer cells to HER2/neu-positive tumors. Nucl. Med. Biol..

[24] Ritchie D., Mileshkin L., Wall D., Bartholeyns J., Thompson M., Coverdale J., Lau E., Wong J., Eu P., Hicks R. (2007). In vivo tracking of macrophage activated killer cells to sites of metastatic ovarian carcinoma. Cancer Immunol. Immunother..

[25] Melder R.J., Brownell A.L., Shoup T.M., Brownell G.L., Jain R.K. (1993). Imaging of activated natural killer cells in mice by positron emission tomography: Preferential uptake in tumors. Cancer Res..

[26] Bhatnagar P., Alauddin M., Bankson J.A., Kirui D., Seifi P., Huls H., Lee D., Babakhani A., Ferrari M., Li K.C. (2014). Tumor Lysing Genetically Engineered T Cells Loaded with Multi-Modal Imaging Agents. Sci. Rep..

[27] Bhatnagar P., Li Z., Choi Y., Guo J., Li F., Lee D.Y., Figliola M., Huls H., Lee D., Zal T. (2013). Imaging of genetically engineered T cells by PET using gold nanoparticles complexed to Copper-64. Integr. Biol..

[28] Singla R., Wall D.M., Anderson S., Zia N., Korte J., Kravets L., McKiernan G., Butler J., Gammilonghi A., Arora J. (2020). First in-human study of in vivo imaging of ex vivo labeled CAR T cells with dual PET-MR. J. Clin. Oncol..

[29] Griessinger C.M., Kehlbach R., Bukala D., Wiehr S., Bantleon R., Cay F., Schmid A., Braumüller H., Fehrenbacher B., Schaller M. (2014). In Vivo Tracking of Th1 Cells by PET Reveals Quantitative and Temporal Distribution and Specific Homing in Lymphatic Tissue. J. Nucl. Med..

[30] Park J.-J., Lee T.-S., Son J.-J., Chun K.-S., Song I.-H., Park Y.-S., Kim K.-I., Lee Y.-J., Kang J.-H. (2012). Comparison of Cell-Labeling Methods with 124I-FIAU and ^64^Cu-PTSM for Cell Tracking Using Chronic Myelogenous Leukemia Cells Expressing HSV1-tk and Firefly Luciferase. Cancer Biother. Radiopharm..

[31] Li Z.-B., Chen K., Wu Z., Wang H., Niu G., Chen X. (2009). ^64^Cu-Labeled PEGylated Polyethylenimine for Cell Trafficking and Tumor Imaging. Mol. Imaging Biol..

[32] Bhargava K.K., Gupta R.K., Nichols K.J., Palestro C.J. (2009). In vitro human leukocyte labeling with ^64^Cu: An intraindividual comparison with 111 In-oxine and ^18^F-FDG. Nucl. Med. Biol..

[33] Adonai N., Nguyen K.N., Walsh J., Iyer M., Toyokuni T., Phelps M.E., McCarthy T., McCarthy D.W., Gambhir S.S. (2002). Ex vivo cell labeling with ^64^Cu-pyruvaldehyde-bis(N4-methylthiosemicarbazone) for imaging cell trafficking in mice with positron-emission tomography. Proc. Natl. Acad. Sci. USA.

[34] Gawne P., Man F., Fonslet J., Radia R., Bordoloi J., Cleveland M., Jimenez-Royo P., Gabizon A., Blower P.J., Long N. (2018). Manganese-52: Applications in cell radiolabelling and liposomal nanomedicine PET imaging using oxine (8-hydroxyquinoline) as an ionophore. Dalton Trans..

[35] Deri M.A., Zeglis B.M., Francesconi L.C., Lewis J.S. (2013). PET imaging with ^89^Zr: From radiochemistry to the clinic. Nucl. Med. Biol..

[36] Charoenphun P., Meszaros L., Chuamsaamarkkee K., Sharif-Paghaleh E., Ballinger J.R., Ferris T.J., Went M., Mullen G.E.D., Blower P.J. (2015). [^89^Zr]Oxinate4 for long-term in vivo cell tracking by positron emission tomography. Eur. J. Nucl. Med. Mol. Imaging.

[37] Lechermann L.M., Manavaki R., Attili B., Lau D., Jarvis L.B., Fryer T.D., Bird N., Aloj L., Patel N., Basu B. (2021). Detection limit of ^89^Zr-labeled T cells for cellular tracking: An in vitro imaging approach using clinical PET/CT and PET/MRI. EJNMMI Res..

[38] Sato N., Wu H., Asiedu K.O., Szajek L.P., Griffiths G.L., Choyke P.L. (2015). ^89^Zr-Oxine Complex PET Cell Imaging in Monitoring Cell-based Therapies. Radiology.

[39] Patrick P.S., Kolluri K.K., Thin M.Z., Edwards A., Sage E.K., Sanderson T., Weil B.D., Dickson J., Lythgoe M.F., Lowdell M. (2020). Lung delivery of MSCs expressing anti-cancer protein TRAIL visualised with ^89^Zr-oxine PET-CT. Stem Cell Res. Ther..

[40] Sato N., Stringaris K., Davidson-Moncada J.K., Reger R., Adler S.S., Dunbar C., Choyke P.L., Childs R.W. (2020). In Vivo Tracking of Adoptively Transferred Natural Killer Cells in Rhesus Macaques Using ^89^Zirconium-Oxine Cell Labeling and PET Imaging. Clin. Cancer Res..

[41] Man F., Lim L., Volpe A., Gabizon A., Shmeeda H., Draper B., Parente-Pereira A.C., Maher J., Blower P., Fruhwirth G.O. (2019). In Vivo PET Tracking of ^89^Zr-Labeled Vγ9Vδ2 T Cells to Mouse Xenograft Breast Tumors Activated with Liposomal Alendronate. Mol. Ther..

[42] Weist M.R., Starr R., Aguilar B., Chea J., Miles J.K., Poku E., Gerdts E., Yang X., Priceman S.J., Forman S.J. (2018). PET of Adoptively Transferred Chimeric Antigen Receptor T Cells with ^89^Zr-Oxine. J. Nucl. Med..

[43] Lee S.H., Soh H., Chung J.H., Cho E.H., Lee S.J., Ju J.M., Sheen J.H., Kim H., Oh S.J., Lee S.-J. (2020). Feasibility of real-time in vivo ^89^Zr-DFO-labeled CAR T-cell trafficking using PET imaging. PLoS ONE.

[44] Agger R., Petersen M.S., Petersen C.C., Hansen S.B., Stødkilde-Jørgensen H., Skands U., Blankenstein T., Andersen T.E., Hulgaard E.F., Jørgensen J.T. (2007). T Cell Homing to Tumors Detected by 3D-coordinated Positron Emission Tomography and Magnetic Resonance Imaging. J. Immunother..

[45] Vedvyas Y., Shevlin E., Zaman M., Min I.M., Amor-Coarasa A., Park S., Park S., Kwon K.-W., Smith T., Luo Y. (2016). Longitudinal PET imaging demonstrates biphasic CAR T cell responses in survivors. JCI Insight.

[46] Park S., Shevlin E., Vedvyas Y., Zaman M., Park S., Hsu Y.-M.S., Min I.M., Jin M.M. (2017). Micromolar affinity CAR T cells to ICAM-1 achieves rapid tumor elimination while avoiding systemic toxicity. Sci. Rep..

[47] Minn I., Huss D.J., Ahn H.-H., Chinn T.M., Park A., Jones J., Brummet M., Rowe S.P., Sysa-Shah P., Du Y. (2019). Imaging CAR T cell therapy with PSMA-targeted positron emission tomography. Sci. Adv..

[48] Volpe A., Lang C., Lim L., Man F., Kurtys E., Ashmore-Harris C., Johnson P., Skourti E., de Rosales R.T., Fruhwirth G.O. (2020). Spatiotemporal PET Imaging Reveals Differences in CAR-T Tumor Retention in Triple-Negative Breast Cancer Models. Mol. Ther..

[49] Moroz M.A., Zhang H., Lee J., Moroz E., Zurita J., Shenker L., Serganova I., Blasberg R., Ponomarev V. (2015). Comparative Analysis of T Cell Imaging with Human Nuclear Reporter Genes. J. Nucl. Med..

[50] Yaghoubi S.S., Jensen M.C., Satyamurthy N., Budhiraja S., Paik D., Czernin J., Gambhir S.S. (2008). Noninvasive detection of therapeutic cytolytic T cells with ^18^F–FHBG PET in a patient with glioma. Nat. Clin. Pract. Oncol..

[51] Keu K.V., Witney T., Yaghoubi S., Rosenberg J., Kurien A., Magnusson R., Williams J., Habte F., Wagner J.R., Forman S. (2017). Reporter gene imaging of targeted T cell immunotherapy in recurrent glioma. Sci. Transl. Med..

[52] Najjar A.M., Manuri P.R., Olivares S., Flores L., Mi T., Huls H., Shpall E.J., Champlin R.E., Turkman N., Paolillo V. (2016). Imaging of Sleeping Beauty-Modified CD19-Specific T Cells Expressing HSV1-Thymidine Kinase by Positron Emission Tomography. Mol. Imaging Biol..

[53] Dobrenkov K., Olszewska M., Likar Y., Shenker L., Gunset G., Cai S., Pillarsetty N.V.K., Hricak H., Sadelain M., Ponomarev V. (2008). Monitoring the Efficacy of Adoptively Transferred Prostate Cancer-Targeted Human T Lymphocytes with PET and Bioluminescence Imaging. J. Nucl. Med..

[54] Sellmyer M.A., Lee I., Hou C., Lieberman B.P., Zeng C., Mankoff D.A., Mach R.H. (2017). Quantitative PET Reporter Gene Imaging with [11C]Trimethoprim. Mol. Ther..

[55] Sellmyer M.A., Richman S.A., Lohith K., Hou C., Weng C.C., Mach R.H., O’Connor R.S., Milone M.C., Farwell M.D. (2020). Imaging CAR T Cell Trafficking with eDFHR as a PET Reporter Gene. Mol. Ther..

[56] Matthews T., Boehme R. (1988). Antiviral Activity and Mechanism of Action of Ganciclovir. Clin. Infect. Dis..

[57] Serganova I., Blasberg R.G. (2019). Molecular Imaging with Reporter Genes: Has Its Promise Been Delivered?. J. Nucl. Med..

[58] Casucci M., Perna S.K., Falcone L., Camisa B., Magnani Z.I., Bernardi M., Crotta A., Tresoldi C., Fleischhauer K., Ponzoni M. (2013). Graft-versus-leukemia Effect of HLA-haploidentical Central-memory T-cells Expanded with Leukemic APCs and Modified With a Suicide Gene. Mol. Ther..

[59] Eissenberg L.G., Rettig M., Dehdashti F., Piwnica-Worms D., DiPersio J.F. (2014). Suicide genes: Monitoring cells in patients with a safety switch. Front. Pharmacol..

[60] Brandt L.J.B., Barnkob M.B., Michaels Y.S., Heiselberg J., Barington T. (2020). Emerging Approaches for Regulation and Control of CAR T Cells: A Mini Review. Front. Immunol..

[61] Yaghoubi S.S., Campbell D.O., Radu C., Czernin J. (2012). Positron Emission Tomography Reporter Genes and Reporter Probes: Gene and Cell Therapy Applications. Theranostics.

[62] Reubi J.C. (2003). Peptide Receptors as Molecular Targets for Cancer Diagnosis and Therapy. Endocr. Rev..

[63] Virgolini I., Ambrosini V., Bomanji J.B., Baum R.P., Fanti S., Gabriel M., Papathanasiou N.D., Pepe G., Oyen W.J., Decristoforo C. (2010). Procedure guidelines for PET/CT tumour imaging with ^68^Ga-DOTA-conjugated peptides: ^68^Ga-DOTA-TOC, ^68^Ga-DOTA-NOC, ^68^Ga-DOTA-TATE. Eur. J. Nucl. Med. Mol. Imaging.

[64] Graham M.M., Gu X., Ginader T., Breheny P., Sunderland J. (2017). ^68^Ga-DOTATOC Imaging of Neuroendocrine Tumors: A Systematic Review and Metaanalysis. J. Nucl. Med..

[65] Zhang H., Moroz M.A., Serganova I., Ku T., Huang R., Vider J., Maecke H.R., Larson S.M., Blasberg R., Smith-Jones P.M. (2011). Imaging Expression of the Human Somatostatin Receptor Subtype-2 Reporter Gene with ^68^Ga-DOTATOC. J. Nucl. Med..

[66] Velikyan I., Sundin A., Sörensen J., Lubberink M., Sandström M., Garske-Román U., Lundqvist H., Granberg D., Eriksson B. (2014). Quantitative and Qualitative Intrapatient Comparison of ^68^Ga-DOTATOC and ^68^Ga-DOTATATE: Net Uptake Rate for Accurate Quantification. J. Nucl. Med..

[67] Yaghoubi S.S., Gambhir S.S. (2006). PET imaging of herpes simplex virus type 1 thymidine kinase (HSV1-tk) or mutant HSV1-sr39tk reporter gene expression in mice and humans using [^18^F]FHBG. Nat. Protoc..

[68] Pauwels E., Cleeren F., Tshibangu T., Koole M., Serdons K., Dekervel J., Van Cutsem E., Verslype C., Van Laere K., Bormans G. (2020). [^18^F]AlF-NOTA-octreotide PET imaging: Biodistribution, dosimetry and first comparison with [^68^Ga]Ga-DOTATATE in neuroendocrine tumour patients. Eur. J. Nucl. Med. Mol. Imaging.

[69] Gomes-Porras M., Cárdenas J.J., Álvarez-Escolá C. (2020). Somatostatin Analogs in Clinical Practice: A Review. Int. J. Mol. Sci..

[70] Strosberg J., El-Haddad G., Wolin E., Hendifar A., Yao J., Chasen B., Mittra E., Kunz P.L., Kulke M.H., Jacene H. (2017). Phase 3 Trial of 177Lu-Dotatate for Midgut Neuroendocrine Tumors. N. Engl. J. Med..

[71] Russell S.J., Carlson S.K. (2012). The Sodium Iodide Symporter (NIS) as an Imaging Reporter for Gene, Viral, and Cell-based Therapies. Curr. Gene Ther..

[72] Ravera S., Reyna-Neyra A., Ferrandino G., Amzel L.M., Carrasco N. (2017). The Sodium/Iodide Symporter (NIS): Molecular Physiology and Preclinical and Clinical Applications. Annu. Rev. Physiol..

[73] Doherty J.O., Jauregui-Osoro M., Brothwood T., Szyszko T., Marsden P.K., Doherty M.J.O., Cook G., Blower P., Lewington V. (2017). ^18^F-Tetrafluoroborate, a PET Probe for Imaging Sodium/Iodide Symporter Expression: Whole-Body Biodistribution, Safety, and Radiation Dosimetry in Thyroid Cancer Patients. J. Nucl. Med..

[74] Diocou S., Volpe A., Jauregui-Osoro M., Boudjemeline M., Chuamsaamarkkee K., Man F., Blower P.J., Ng T., Mullen G.E.D., Fruhwirth G.O. (2017). [^18^F]tetrafluoroborate-PET/CT enables sensitive tumor and metastasis in vivo imaging in a sodium iodide symporter-expressing tumor model. Sci. Rep..

[75] Vandergaast R., Khongwichit S., Jiang H., DeGrado T.R., Peng K.-W., Smith D.R., Russell S.J., Suksanpaisan L. (2020). Enhanced noninvasive imaging of oncology models using the NIS reporter gene and bioluminescence imaging. Cancer Gene Ther..

[76] Schwarzenboeck S.M., Rauscher I., Bluemel C., Fendler W.P., Rowe S.P., Pomper M.G., Asfhar-Oromieh A., Herrmann K., Eiber M. (2017). PSMA Ligands for PET Imaging of Prostate Cancer. J. Nucl. Med..

[77] Pacholczyk T., Blakely R.D., Amara S. (1991). Expression cloning of a cocaine-and antidepressant-sensitive human noradrenaline transporter. Nature.

[78] Ashmore-Harris C., Iafrate M., Saleem A., Fruhwirth G.O. (2020). Non-invasive Reporter Gene Imaging of Cell Therapies, including T Cells and Stem Cells. Mol. Ther..

[79] Dammes N., Peer D. (2020). Monoclonal antibody-based molecular imaging strategies and theranostic opportunities. Theranostics.

[80] Liu Z., Li Z. (2014). Molecular Imaging in Tracking Tumor-Specific Cytotoxic T Lymphocytes (CTLs). Theranostics.

[81] Kobayashi H., Choyke P.L., Ogawa M. (2016). Monoclonal antibody-based optical molecular imaging probes; considerations and caveats in chemistry, biology and pharmacology. Curr. Opin. Chem. Biol..

[82] Bolkestein M., De Blois E., Koelewijn S.J., Eggermont A.M., Grosveld F., De Jong M., Koning G.A. (2015). Investigation of Factors Determining the Enhanced Permeability and Retention Effect in Subcutaneous Xenografts. J. Nucl. Med..

[83] Fay R., Holland J.P. (2019). The Impact of Emerging Bioconjugation Chemistries on Radiopharmaceuticals. J. Nucl. Med..

[84] Sarko D., Eisenhut M., Haberkorn U., Mier W. (2012). Bifunctional chelators in the design and application of radiopharmaceuticals for oncological diseases. Curr. Med. Chem..

[85] Van Dongen G.A.M.S., Visser G.W.M., Lub-de Hooge M.N., de Vries E.G., Perk L.R. (2007). Immuno-PET: A Navigator in Monoclonal Antibody Development and Applications. Oncologist.

[86] Tavaré R., Escuin-Ordinas H., Mok S., McCracken M.N., Zettlitz K.A., Salazar F.B., Witte O.N., Ribas A., Wu A.M. (2016). An Effective Immuno-PET Imaging Method to Monitor CD8-Dependent Responses to Immunotherapy. Cancer Res..

[87] Pandit-Taskar N., Postow M.A., Hellmann M.D., Harding J.J., Barker C.A., O’Donoghue J.A., Ziolkowska M., Ruan S., Lyashchenko S.K., Tsai F. (2020). First-in-Humans Imaging with ^89^Zr-Df-IAB22M2C Anti-CD8 Minibody in Patients with Solid Malignancies: Preliminary Pharmacokinetics, Biodistribution, and Lesion Targeting. J. Nucl. Med..

[88] Parisi G., Saco J.D., Salazar F.B., Tsoi J., Krystofinski P., Saus C.P., Zhang R., Zhou J., Cheung-Lau G.C., Garcia A.J. (2020). Persistence of adoptively transferred T cells with a kinetically engineered IL-2 receptor agonist. Nat. Commun..

[89] Mall S., Yusufi N., Wagner R., Klar R., Bianchi H., Steiger K., Straub M., Audehm S., Laitinen I., Aichler M. (2016). Immuno-PET Imaging of Engineered Human T Cells in Tumors. Cancer Res..

[90] Yusufi N., Mall S., Bianchi H.D.O., Steiger K., Reder S., Klar R., Audehm S., Mustafa M., Nekolla S., Peschel C. (2017). In-depth Characterization of a TCR-specific Tracer for Sensitive Detection of Tumor-directed Transgenic T Cells by Immuno-PET. Theranostics.

[91] Davey A.S., Call M.E., Call M.J. (2021). The influence of chimeric antigen receptor structural domains on clinical outcomes and associated toxicities. Cancers.

[92] Shaffer T.M., Aalipour A., Schürch C.M., Gambhir S.S. (2020). PET Imaging of the Natural Killer Cell Activation Receptor NKp30. J. Nucl. Med..

[93] Simonetta F., Alam I.S., Lohmeyer J.K., Sahaf B., Good Z., Chen W., Xiao Z., Hirai T., Scheller L., Engels M.P. (2020). Molecular Imaging of Chimeric Antigen Receptor T Cells By ICOS-Immunopet. Blood.

[94] Sharma P., Allison J.P. (2015). Immune Checkpoint Targeting in Cancer Therapy: Toward Combination Strategies with Curative Potential. Cell.

[95] Bensch F., Van Der Veen E.L., Lub-de Hooge M.N., Jorritsma-Smit A., Boellaard R., Kok I.C., Oosting S.F., Schröder C.P., Hiltermann T.J.N., Van Der Wekken A.J. (2018). ^89^Zr-atezolizumab imaging as a non-invasive approach to assess clinical response to PD-L1 blockade in cancer. Nat. Med..

[96] Niemeijer A.N., Leung D., Huisman M.C., Bahce I., Hoekstra O.S., Van Dongen G.A.M.S., Boellaard R., Du S., Hayes W., Smith R. (2018). Whole body PD-1 and PD-L1 positron emission tomography in patients with non-small-cell lung cancer. Nat. Commun..

[97] Kelly M.P., Tavare R., Giurleo J.T., Makonnen S., Hickey C., Danton M.A., Arnold T.C., Ma D., Dai J., Pei J. (2018). Abstract 3033: Immuno-PET detection of LAG-3 expressing intratumoral lymphocytes using the zirconium-89 radiolabeled fully human anti-LAG-3 antibody REGN3767. Tumor Biol..

[98] Natarajan A., Mayer A.T., Reeves R.E., Nagamine C.M., Gambhir S.S. (2017). Development of Novel ImmunoPET Tracers to Image Human PD-1 Checkpoint Expression on Tumor-Infiltrating Lymphocytes in a Humanized Mouse Model. Mol. Imaging Biol..

[99] England C.G., Jiang D., Ehlerding E.B., Rekoske B.T., Ellison P.A., Hernandez R., Barnhart T.E., McNeel D.G., Huang P., Cai W. (2018). ^89^Zr-labeled nivolumab for imaging of T-cell infiltration in a humanized murine model of lung cancer. Eur. J. Nucl. Med. Mol. Imaging.

[100] Maute R.L., Gordon S.R., Mayer A.T., McCracken M.N., Natarajan A., Ring N.G., Kimura R., Tsai J.M., Manglik A., Kruse A.C. (2015). Engineering high-affinity PD-1 variants for optimized immunotherapy and immuno-PET imaging. Proc. Natl. Acad. Sci. USA.

[101] Larimer B.M., Wehrenberg-Klee E., Caraballo A., Mahmood U. (2016). Quantitative CD3 PET Imaging Predicts Tumor Growth Response to Anti-CTLA-4 Therapy. J. Nucl. Med..

[102] Beckford Vera D.R., Smith C.C., Bixby L.M., Glatt D.M., Dunn S.S., Saito R., Kim W.Y., Serody J.S., Vincent B.G., Parrott M.C. (2018). Immuno-PET imaging of tumor-infiltrating lymphocytes using zirconium-89 radiolabeled anti-CD3 antibody in immune-competent mice bearing syngeneic tumors. PLoS ONE.

[103] Kristensen L.K., Fröhlich C., Christensen C., Melander M.C., Poulsen T.T., Galler G.R., Lantto J., Horak I.D., Kragh M., Nielsen C.H. (2019). CD4^+^ and CD8a^+^ PET imaging predicts response to novel PD-1 checkpoint inhibitor: Studies of Sym021 in syngeneic mouse cancer models. Theranostics.

[104] Nagle V.L., Henry K.E., Hertz C.A.J., Graham M.S., Campos C., Parada L.F., Pandit-Taskar N., Schietinger A., Mellinghoff I.K., Lewis J.S. (2021). Imaging Tumor-Infiltrating Lymphocytes in Brain Tumors with [^64^Cu]Cu-NOTA-anti-CD8 PET. Clin. Cancer Res..

[105] Alam I.S., Mayer A.T., Sagiv-Barfi I., Wang K., Vermesh O., Czerwinski D.K., Johnson E., James M.L., Levy R., Gambhir S.S. (2018). Imaging activated T cells predicts response to cancer vaccines. J. Clin. Investig..

[106] Ehlerding E.B., Lee H.J., Jiang D., Ferreira C.A., Zahm C.D., Huang P., Engle J.W., McNeel D.G., Cai W. (2019). Antibody and fragment-based PET imaging of CTLA-4^+^ T-cells in humanized mouse models. Am. J. Cancer Res..

[107] Partridge M., Spinelli A.E., Ryder W., Hindorf C. (2006). The effect of β^+^ energy on performance of a small animal PET camera. Nucl. Instrum. Methods Phys. Res. Sect. A Accel. Spectrometers Detect. Assoc. Equip..

